# All-cause mortality and three aspects of social relationships: an eight-year follow-up of older adults from one entire Korean village

**DOI:** 10.1038/s41598-020-80684-5

**Published:** 2021-01-11

**Authors:** Yoosik Youm, Ekaterina Baldina, Jiwon Baek

**Affiliations:** 1 Department of Sociology, Yonsei University, Seoul, 03722 Republic of Korea; 2 Institute of Health Policy and Management, Medical Research Center, Seoul National University, Seoul, 03080 Republic of Korea

**Keywords:** Epidemiology, Epidemiology, Risk factors, Human behaviour

## Abstract

Various aspects of social relationships have been examined as risk factors for mortality. In particular, most research has focused on either loneliness or social disengagement. We aimed to extend the current research by adding a group-level segregation measure utilizing the whole social network of one entire village in South Korea. The analyses were based on the Korean Social Life, Health and Aging Project data collected over eight years across five waves. Of the 679 old adults who participated throughout the entire project (to wave 5), 63 were confirmed as deceased. All three aspects of social relationships examined, loneliness, social disengagement, and group-level segregation, were associated with mortality in the traditional Cox proportional hazard model without considering health-related time-varying covariates. However, a Cox marginal structural model, a counterfactual statistical measure that is designed to control for censoring bias due to sample attrition over the eight years and time-varying confounding variables, revealed that only group-level segregation was associated with mortality. Our results strongly suggest that more attention is needed on group-level segregation for mortality studies, as well as on well-known individual-level risk factors, including social disengagement and loneliness. All methods were carried out in accordance with relevant guidelines and regulations.

## Introduction

Various social relationships have been examined as risk factors for an increased mortality rate among older adults^1–8^. Notably, two related, but distinctive, aspects of social relationships have been extensively studied. First, perceived loneliness is considered a measure of perceived isolation, indicating a discrepancy between an individual’s desired and actual social connections^9^. Researchers have found that loneliness is universal, to some extent, across all age groups and has a devastatingly negative effect on people’s health statuses^10–12^. One meta-analysis involving 77,220 participants found that lonely people have a 22% higher risk of mortality^6^. Prospective studies have shown that loneliness is related to mortality through reductions in an individual’s physical functioning^13^, an increase in depression^14^, decreased health statuses^15^, and a greater risk for developing chronic diseases^16^.

Social disengagement is defined as a withdrawal from social life and activities with others^9,17–20^. Aspects of social engagement, such as organizational attendance^16^, religious participation^9,21,22^, volunteering activity^23–25^, and communication with friends^1,26–28^, are all predictors of a lowered mortality risk. Participating in various social activities can lower a person’s mortality risk through a series of physiological and psychological pathways^29–31^. Previous research has discovered that social activities are linked to diverse health statuses, including cognitive health^32^, depression^33^, and physical health^34^. Additionally, social engagement has been shown to provide a basis for the initiation of social norms and, consequently, is an encouraging factor in the adoption of healthier lifestyles, as well as the promotion of medication adherence^35,36^. It also provides a channel for the communication of health-related information while increasing the social pressure and motivation to engage in health-friendly behaviors while enhancing a person’s overall health literacy^37,38^.

**Figure 1:**
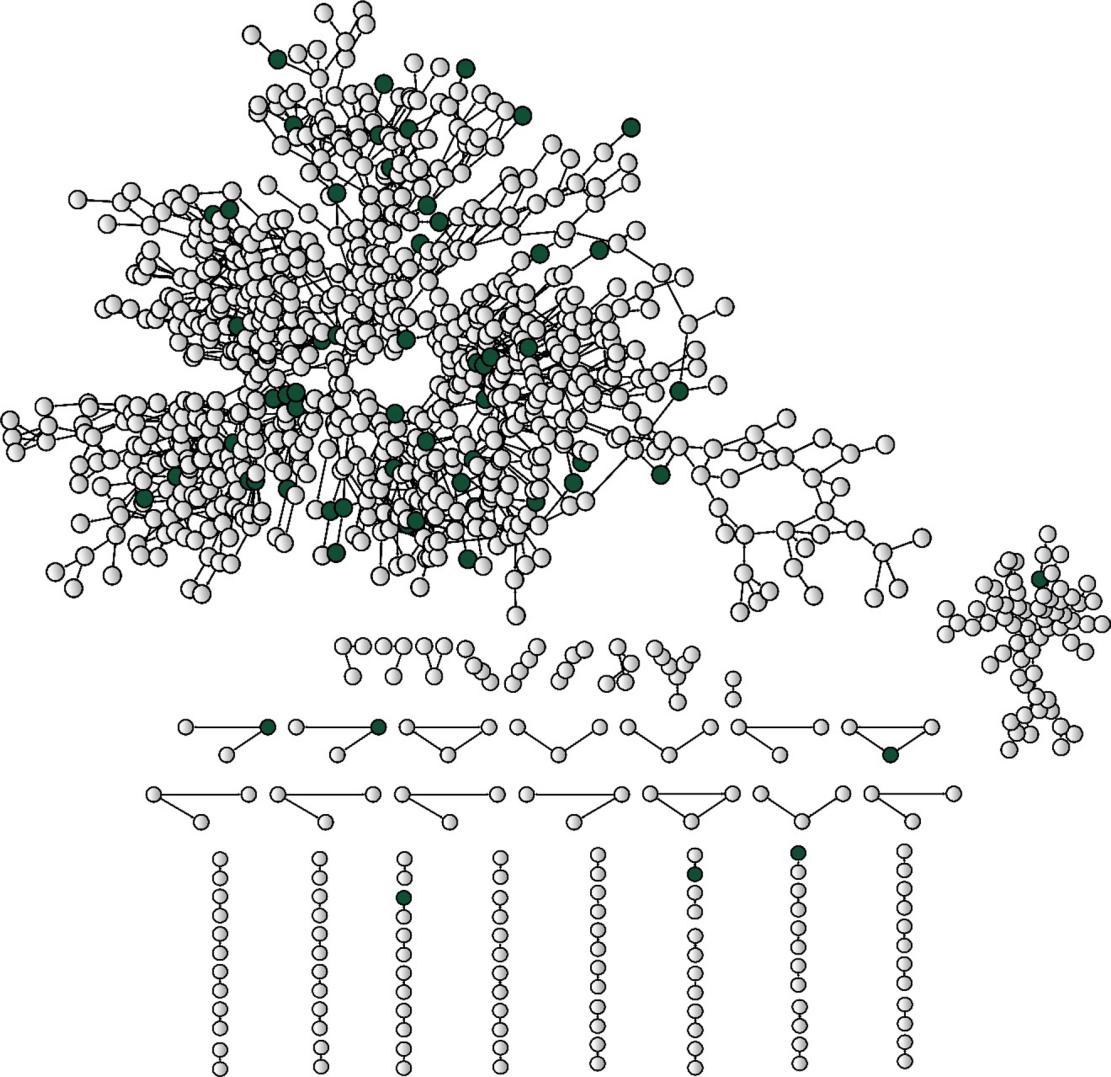
The complete social network of village K (n = 995) in 2011. Dark green nodes represent deceased respondents (n = 63). Lines between nodes represent social relationships between the residents.

In addition to loneliness and social disengagement, we included a third dimension of social relationships that is a risk factor for mortality: group-level segregation. This study defines group-level segregated people as those who belong to a social group with a relatively small diameter. In social network analyses, the diameter of a group refers to the length of the largest geodesic path within that same group. For example, if a friendship circle has a diameter of two, every person in that group is either each other’s friend or a second-degree association of a friend (i.e., a friend’s friend). Group-level segregation measures a different dimension of social relationships from that of loneliness or disengagement— people who are group-level segregated may have many friends, but even if they were to extend the degree of connections as far as possible, the total number of social ties they are able to reach would be small and, thus, they are segregated as a group. In some communities, people are able to engage in various social activities with their close friends and, thus, do not feel lonely; however, if all of their friends, as a group, form a small clique that is segregated from the rest of the community, they are then classified as group-level segregated. To measure group-level segregation, we needed the complete social network of the entire village under study. Figure 1 shows the complete social network of village K in South Korea.

There have been other aspects of social relationships that are seemingly similar to the concept of group-level segregation and widely examined as risk factors for mortality. Instead of providing an extensive review of all seemingly related measures, we discuss three most widely used social relationships for the comparison purpose. Bridging social capital refers to the social connections between two people who won’t be connected otherwise and has been confirmed as a risk factor for mortality in many studies^39–42^. Another related concept, social exclusion, usually examined the excluded and isolated people such as homeless, bullied adolescents and living-alone older adults and their mortality rates^43–45^. Also, social network index developed by Berman and Syme^2^ has been widely examined by numerous studies^7,8,46^. Social Network Index (SNI) usually measured social ties as a composite index along four dimensions: (1) tie with a spouse, (2) contacts with close friends and relatives, (3) membership in a church group, (4) membership in other types of groups.

Although group-level segregation is related to these concepts, there is one very distinctive difference: the unit of analysis. All three related concepts are measured and examined at the individual level^47^ while group-level segregation measures above and beyond individual-level social relationship. It is quite possible that a group of residents in a village is not excluded, has rich bridging social capital and maintain a high score of SNI but is still segregated as a group from the whole community. In other words, people sometimes manage to keep good and rich social relationships within their social group but are segregated from other social groups. One good example would be racial segregation: a certain racial/ ethnic group can enjoy rich social relationships within its own racial/ethnic group, but its social ties do not extend outside the racial/ethnic group. This type of collective-level social relationships has largely been ignored not because it is inconsequential but because of the lack of empirical data. Our data allowed us to examine group-level segregation since it included a complete social network of one entire village.

## Results

The characteristics of the study sample in terms of the three aspects of social relationships are summarized in Table 1. The average age of the sample included in the analyses was 73.9 years, and 57.4% of the respondents were women. There were apparent discrepancies based on social relationship status. In general, people who were lonely or disengaged were older, less educated and more likely to be women, unmarried, and poorer than those who were not. They were also more depressed and in poorer physical and cognitive health. Segregated respondents were more educated and less likely to be married than people who were not segregated; they were also more depressed and had slightly worse cognitive health than those who were not segregated. The results contained in Table 1 strongly imply the need to control many time-related covariates to examine the causal effects of the three aspects of social relationships on mortality, since they were systematically associated with several sociodemographic and health variables.

**Table 1:** Characteristics of the sample stratified by levels of loneliness, social disengagement, and group-level segregation at the censor/failure time (N = 679).

Variables		Total	Not lonely	Lonely	*p* Value	Engaged	Disengaged	*p* Value	Not segregated	Segregated	*p* Value
		N = 679	N = 497	N = 182		N = 571	N = 108		N = 547	N = 132	
Age		73.9 (7.5)	72.8 (7.0)	77.0 (7.8)	< 0.001	73.0 (7.0)	78.9 (7.9)	< 0.001	74.1 (7.3)	73.3 (8.1)	0.26
Education	< High school	479 (70.5%)	329 (66.2%)	150 (82.4%)	< 0.001	382 (66.9%)	97 (89.8%)	< 0.001	400 (73.1%)	79 (59.8%)	0.003
	≥ High school	200 (29.5%)	168 (33.8%)	32 (17.6%)		189 (33.1%)	11 (10.2%)		147 (26.9%)	53 (40.2%)	
Sex	Male	289 (42.6%)	223 (44.9%)	66 (36.3%)	0.045	256 (44.8%)	33 (30.6%)	0.006	238 (43.5%)	51 (38.6%)	0.31
	Female	390 (57.4%)	274 (55.1%)	116 (63.7%)		315 (55.2%)	75 (69.4%)		309 (56.5%)	81 (61.4%)	
Living with spouse	No	164 (24.2%)	81 (16.3%)	83 (45.6%)	< 0.001	112 (19.6%)	52 (48.1%)	< 0.001	122 (22.3%)	42 (31.8%)	0.022
	Yes	515 (75.8%)	416 (83.7%)	99 (54.4%)		459 (80.4%)	56 (51.9%)		425 (77.7%)	90 (68.2%)	
Yearly income	< $10,000	311 (45.8%)	202 (40.6%)	109 (59.9%)	< 0.001	234 (41.0%)	77 (71.3%)	< 0.001	253 (46.3%)	58 (43.9%)	0.63
	≥ $10,000	368 (54.2%)	295 (59.4%)	73 (40.1%)		337 (59.0%)	31 (28.7%)		294 (53.7%)	74 (56.1%)	
Smoking	No	620 (91.3%)	455 (91.5%)	165 (90.7%)	0.72	522 (91.4%)	98 (90.7%)	0.82	498 (91.0%)	122 (92.4%)	0.61
	Yes	59 (8.7%)	42 (8.5%)	17 (9.3%)		49 (8.6%)	10 (9.3%)		49 (9.0%)	10 (7.6%)	
Drinking	No	553 (81.4%)	399 (80.3%)	154 (84.6%)	0.20	452 (79.2%)	101 (93.5%)	< 0.001	438 (80.1%)	115 (87.1%)	0.062
	Yes	126 (18.6%)	98 (19.7%)	28 (15.4%)		119 (20.8%)	7 (6.5%)		109 (19.9%)	17 (12.9%)	
Depression^†^		− 0.1 (0.6)	− 0.3 (0.3)	0.6 (0.8)	< 0.001	− 0.1 (0.6)	0.3 (0.8)	< 0.001	− 0.1 (0.6)	0.1 (0.7)	0.005
Physical health		44.5 (10.9)	46.2 (10.2)	39.8 (11.5)	< 0.001	46.0 (10.1)	36.8 (11.8)	< 0.001	44.3 (10.8)	45.1 (11.6)	0.48
Cognitive health		24.1 (5.0)	24.8 (4.4)	22.2 (6.0)	< 0.001	24.9 (4.1)	19.5 (6.7)	< 0.001	24.3 (4.6)	23.3 (6.6)	0.046
Comorbidity		2.6 (0.8)	2.6 (0.8)	2.8 (0.9)	0.003	2.6 (0.8)	2.6 (0.7)	0.88	2.6 (0.8)	2.6 (0.8)	0.70

Numbers represent the mean (standard deviation) for continuous measures and cell frequency (column %) for binary measures.

^†^The CES-D score for depression was standardized.

**Figure 2:**
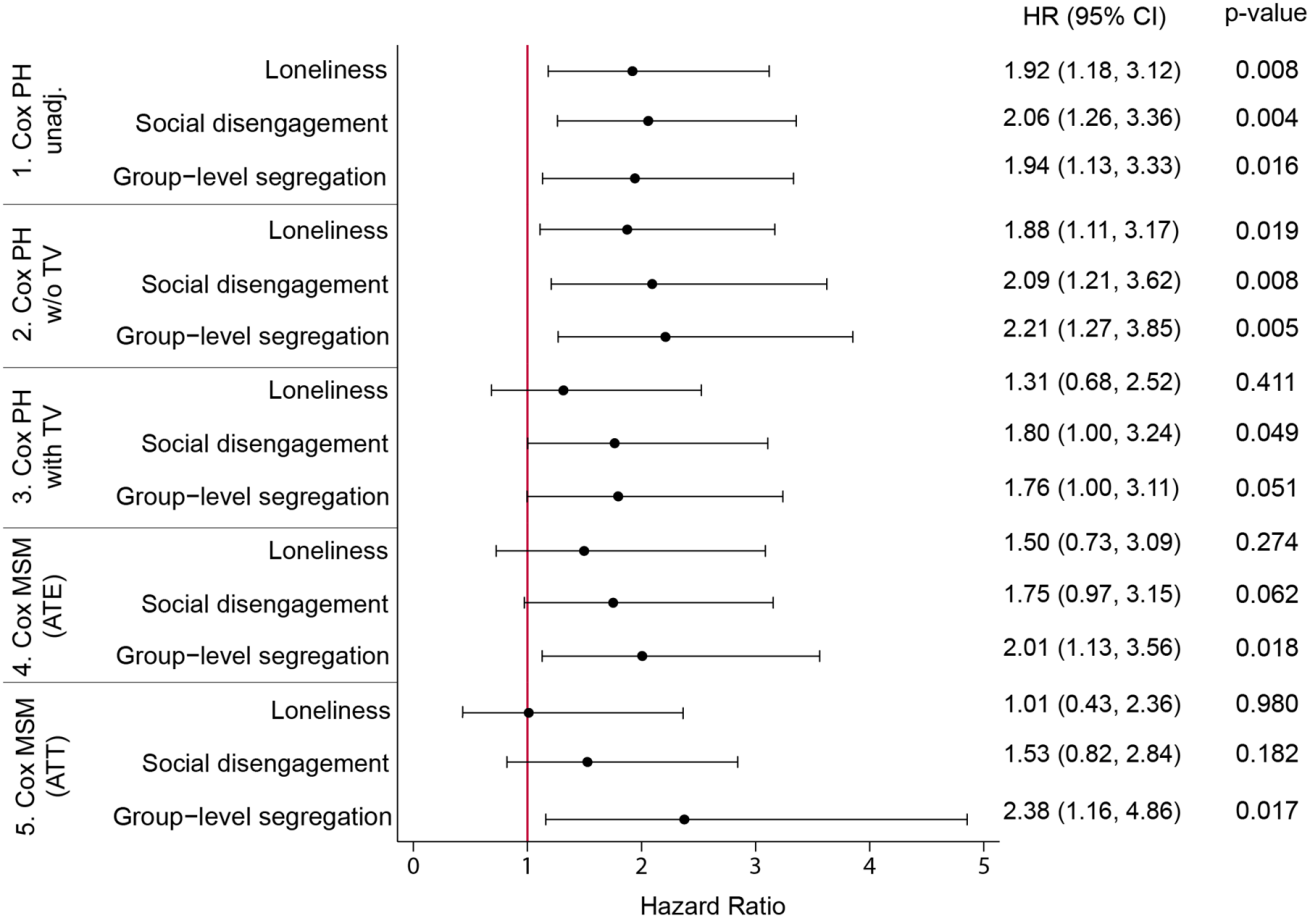
The hazard ratios of loneliness, social disengagement and group-level segregation from Cox PH regression models and Cox MSM models (n = 679). Model 1. Cox PH unadjusted model; no control variables included; Model 2. Cox PH w/o TV: education, sex, loneliness (baseline), social disengagement (baseline), segregation (baseline), living with spouse (baseline), yearly income (baseline), smoking (baseline), drinking (baseline), depression (baseline), physical health (baseline), cognitive health (baseline), comorbidity (baseline); Model 3. Cox PH with TV: Model 2 covariates + loneliness, social disengagement, segregation, living with spouse, yearly income, smoking, drinking, depression, physical health, cognitive health, comorbidity – covariates changing over time; Model 4. Cox MSM (ATE): education, sex, loneliness (baseline), social disengagement (baseline), segregation (baseline), living with spouse (baseline), yearly income (baseline), smoking (baseline), drinking (baseline), depression (baseline), physical health (baseline), cognitive health (baseline), comorbidity (baseline); Model 5. Cox MSM (ATT): education, sex, loneliness (baseline), social disengagement (baseline), segregation (baseline), living with spouse (baseline), yearly income (baseline), smoking (baseline), drinking (baseline), depression (baseline), physical health (baseline), cognitive health (baseline), comorbidity (baseline). Exponentiated coefficients; HR-hazard ratios; TV-time-varying covariates; ATE-average treatment effects; ATT-average treatment effects of treated; 95% confidence intervals in parentheses.

Figure 2 illustrates the effects of each of the three social relationships on all-cause mortality among older adults throughout the eight-year period. Lonely people were almost twice as likely to die when we estimated the effect without controlling for any covariates in a traditional Cox proportional hazard model. They were still 1.88 times more likely to die when we controlled for time-constant covariates. However, the effect lost most of its statistical significance once we controlled for time-varying covariates, yielding a *p* value of 0.411. The two Cox Marginal Structural Model (MSM)s also revealed that loneliness had no causal effect once we adjusted for biases from reverse causation based on inverse probability weighting.

Similar results were found for social disengagement. Socially disengaged older adults were two times more likely to die when we did not include any covariates in a Cox Proportional Hazard (PH) model. Even after adjusting for relevant time-constant covariates, they were twice as likely to pass away. Once we added the time-variant covariates, the effect lost much of its statistical significance, although it remained marginally significant (95% CI: 1.00; 3.24). However, two Cox MSMs showed that we could not confirm causality in the relationship between social disengagement and death.

Group-level segregation, however, showed persistent effects in almost all models. Group-level segregated respondents were approximately 80% (95% CI: 1.00; 3.11) more likely to die according to traditional Cox PH models, even after we controlled for time-varying covariates. Furthermore, they were approximately twice as likely to die when we tried to estimate causal effects by assigning inverse probability weights to adjust for time-varying confounders. The average treatment effect, measured as the hazard ratio, for all respondents was 2.01; the average treatment effect on the treated, in other words, the difference between people who were actually segregated and people who were not hypothetically segregated in the counterfactual situation, was 2.38. The accuracy of the estimates from Cox MSMs is very dependent on the goodness-of-fit of the models. For example, since we tried to remove the statistically significant difference in cognitive health between segregated and nonsegregated people, by assigning inverse probability weight (IPW) for each respondent to estimate causal effects of segregation, it was essential to confirm the independence of cognitive health and segregation in the Cox MSM. To do so, we conducted extensive balance checks by computing weights and comparing the distribution of control variables before and after weighting with a mean standardized difference. Balance checks for the Cox MSMs for each of the three social relationships are summarized in the supplementary material (Tables S5a ~ S7b).

## Discussion

The results can be summarized as follows: (1) all three aspects of social relationships were good predictors of all-cause mortality with small *p* values in older adults over the eight-year study period when controlling for confounding baseline factors; (2) loneliness and social disengagement lost their significance once we controlled for time-variant confounders; and (3) the association of group-level segregation with mortality was robust and did not disappear, even after controlling for time-variant confounders including comorbid conditions and physical and cognitive health, in a Cox MSM that aimed to minimize bias from such confounders.

Loneliness, one of the most studied aspects of social relationships to predict mortality, lost most of its statistical significance once we controlled for time-varying confounders. This implies that diverse health statuses and loneliness would have a reciprocal association in terms of mortality; this result is consistent with previous longitudinal studies^13–15,48,49^. Using a nationally representative American sample from the Health and Retirement Study (HRS), one study^15^ revealed that loneliness increased all-cause mortality risk over six years. However, this association was no longer statistically significant when baseline depressive symptoms, self-rated health, and functional limitations were added together, implying reciprocal prospective effects of loneliness and health statuses. Moreover, a study of older Chinese adults^49^ showed that emotional health and functional limitations partially mediated the effect of loneliness on mortality. Another study, based on data from the Alameda County Health and Ways of Living Study^14^, also found that the association between loneliness and mortality was largely explained by physical activity and depression.

A very similar pattern was revealed regarding social disengagement: its statistical significance substantially decreased when we introduced time-variant confounders into the model. It is well known that engagement in social activities helps to maintain good health and high life satisfaction^50–52^, prevents the deterioration of physical functioning^2,53,54^, and reduces the risk of disability development^55^. Social engagement has also been known to help maintain cognitive function^56,57^ and decrease the risk of dementia^58^, previously found to be associated with institutionalization and mortality^59^. Similar to loneliness, social disengagement may interact with physical health in a reciprocal way, which eventually leads to mortality^29,60^.

The results related to loneliness and disengagement have critical implications for the development of effective preventive strategies to decrease mortality rates among older adults, as they illustrate the mechanisms through which loneliness or disengagement could result in death. The effect of social disengagement reduced its statistical significance as soon as we included physical health status in the equation and lost its significance in the Cox MSMs. Loneliness lost its statistical significance only after depression was controlled for. These results strongly imply that the social disengagement effect could be explained by a lack of physical health, while loneliness tends to induce death mainly through depression among older adults (see supplementary material, Tables S8 and S9). This kind of information will be critical for developing customized and effective interventions. For example, public health agents can monitor and support older adults who feel both lonely and depressed in their target areas.

To our knowledge, this is the first study to directly examine group-level segregation as a risk factor for mortality using complete social network data. The fact that group-level segregation was not fully mediated by other time-varying confounders in the Cox MSMs, unlike loneliness and disengagement, suggests that it might have unique pathways to mortality. As discussed earlier, group-level segregated respondents in our sample were more educated, while they were also more likely to be depressed and have worse cognitive health. Furthermore, their residential duration (32.2 years) was shorter than that of other responders (58.2 years). We believe that many of the group-level segregated respondents moved to this tight knit, traditional rural village from urban areas, likely after their retirement. They were more educated than people who were firmly embedded in the village, but they were segregated as a group and showed a higher level of depression. Even if they could manage the rich social relations among themselves, their social relationships remained within their small groups and did not reach the whole village. In this sense, group-level segregation could be related to the concept of network scope. A 15-year prospective study showed social network scope, measure of the number of different domains in which a person keeps social ties, was a consistent predictor for mortality among people who had incidences of Ischemic heart disease, cancer, and stroke^61^. Domains identified were family, friends, neighborhoods, and broader community organizations. The social domains of older adults who were group-level segregated in our sample could be limited to family and friends only.

We conjecture that group-level segregation might be related to mortality through three major pathways. First, they would have difficulty obtaining useful and timely health-related information since their social ties and thus their sources of information are segregated. Social networks provide significant information about the monitoring and diagnosis of diseases^62,63^, and limited information and resource exchange in group-level segregated older adults may make them more vulnerable and susceptible to various health problems^64^. For example, group-level segregated respondents would have been excluded from various village-wide health-related services or programs, such as free regular medical screenings provided by the government^65,66^. We suppose that group-level segregated respondents in our analysis may have had difficulties accessing valuable health-related information due to the absence of ties to the whole village, and their immediate contacts may have failed to provide them with useful information when needed^67,68^.

Second, group-level segregation might be linked to the lack of a sense of belonginess, which could then be implicated in poor health and even mortality. Even when group-level segregated respondents obtain valuable health-related information in time, they might not want to participate in it since they do not feel they are legitimate members of the village. We believe the segregated older adults were aware of the segregation, and they would therefore have had difficulty securing a sense of belonging in the whole community. A large body of evidence suggests that people are happier and healthier when they experience social belonging^69–72^. An earlier study showed that a sense of belonging and trusting neighborly relationships were vital elements of the support system of older adults^73^. Indeed, a sense of belonging, as one of the basic human needs, has been shown to enhance the psychological well-being of individuals and reduce suicidal ideation and depression symptoms^74–78^; previous studies have shown that a sense of belonging contributed to the survival of community-dwelling older adults through psychological pathways^79,80^. The sense of belonging to the community among segregated Indian Americans also explained individual differences in mental health and mortality by suicide^81–83^. Also, studies on minorities, marginalized and socially vulnerable groups showed that the lack of sense of belonging associated with stress^84,85^. Cohen and Wills (1985) also took social companionship (belongingness) as one of four social resources that can operate as stress buffers along with other types of social resources including esteem support (or emotional support), informational support and instrumental support^86^. Considering many studies revealed that stress could lead to deteriorating health through endocrine system^87–90^, we believe group-level segregation could lead to death by either high-level exposure of stress or the lack of stress buffers. Thus, a personal sense of belonging to the community plays an important role in suppressing the negative effects of the social environment on health. In contrast, the absence of a sense of belonging and the lack of village-wide emotional support could increase the mortality rate of segregated people.

Last, group-level segregation could mean not only having a lack of sense of belonging but also being the target of hostility and disrespect. It is well known that segregated African Americans experience higher rates of cancer occurrence, elevated risk of cardiovascular disease occurrence, respiratory function and illness, and have lower self-rated health, suggesting that living in adverse environments imposes an immune burden and potential compromise in the body’s normal defenses^91–95^. For this reason, racial segregation has contributed to the development of multiple chronic conditions and outcomes, leading to widening health disparities between races in the USA^96–98^. Social segregation that poses a constant threat of hostility, denigration, and disrespect promotes exposure to allostatic load and stress, which results in chronically high levels of inflammation^99,100^, and the amount of exposure to racial segregation successfully predicted inflammation among African Americans^101,102^. It is widely known that in a Korean rural village where strong cohesive communities are very active, small, segregated groups face severe hostility, disrespect, and even discrimination.

Although our study benefits from a unique longitudinal data set that contains the complete social network of an entire village, it also has one major limitation worthy of discussion here. Our sample is restricted to a traditional rural village in Korea, and it would be inappropriate to generalize our findings to other areas or countries without due consideration. The association of social relationships with mortality should be contingent on diverse factors such as culture, cause of deaths, kind of supports, and gender. For example, the British Whitehall II cohort study revealed that among men, network score predicted all-cause and cardiovascular mortality but not cancer mortality^103^. Another research based on the German Heinz Nixdorf Recall Study^104^ showed that perceiving a lack of financial support predicted incident cardiovascular events while social isolation was associated with all-cause mortality. A 15-year prospective study showed that social network scope, a measure of the number of different domains in which a person has social contacts, was a consistent predictor for mortality^61^. In this regard, future studies with detailed measures of different aspects of social relationships and various causes of deaths in urban areas or other countries would be invaluable.

For this reason, we do not believe that loneliness or social disengagement could be dropped out of the list of the crucial risk factors for mortality. For example, the concept of loneliness itself is quite ubiquitous across cultures, although there could be substantial differences in how older adults could understand it^105^. Our study results should emphasize a need for new attention to group-level segregation rather than to weakened awareness of loneliness or social disengagement.

This new research direction would be especially valuable if there exist a certain group of people who maintain good and rich individual-level social relationships and but are segregated as a group and thus vulnerable to death. Considering most public health strategies for mortality have been based on individual-level risk factors, we believe this new line of studies could shed a new light on public health strategies to improve longevity.

## Materials and methods

### Study sample and mortality

The data, including those on the complete social network of the entire village, were obtained from the Korean Social Life, Health and Aging Project (KSHAP), which was collected across five waves: wave 1 (2011), wave 2 (2012), wave 3 (2014–2015), wave 4 (2015–2016), and wave 5 (2018–2019)^106^. This village, village K, is a typical, rural Korean village where farming is the main industry. Village K is located on the north side of Ganghwa island and has an area of about 6,500 acres. With the aid of the public officers of village K and a pilot study, a total of 860 people aged 60 or older and their spouses were identified as the KSHAP population. About 67 percent of our respondents were working and 88 percent of them were active in farming^106^. This project has collected data on the complete social networks of and various biomarkers in older adults (aged 60 years or older) and their spouses since 2011. The face-to-face survey was completed with 814 out of the 860 target residents during wave 1, with a response rate of 94.7%. Among the 679 people who were successfully followed up through wave 5, 63 were confirmed as deceased. The respondents were educated on the nature of the survey, with informed written consent being obtained before they completed the survey. This study was approved by both the institutional review board of Yonsei University (YUIRB-2011–012-01 in 2011; 1,040,917–201,505-SB-152–05 in 2014; 7,001,988–201,806-HRBR-244–04 in 2016; 7,001,988–201,812-h-505–02 in 2018) and the Yonsei University Health System, Severance Hospital (4–2012-0172 in 2012). The outcome measure included any deaths up until December 2018 that were reported during the follow-ups and that were confirmed by the close acquaintances such as spouses or relatives of the deceased during each wave. Unfortunately, we were not able to match the information with death certificates since we didn’t obtain written permissions in advance from the deceased to secure death certificates. During the eight-year follow-up, the mean follow-up period was 5.61 years, with a standard deviation of 1.68 years. Respondents’ ages were used as the time scale in the Cox models, as is commonly practiced, with their ages ranging from 42 to 98^107,108^.

### Loneliness

An item from the Center for Epidemiologic Studies Depression Scale (CES-D) was used to measure the perceived loneliness of participants^9,109^. The item from the CES-D was correlated with a validated measure from the University of California, Los Angeles (UCLA) loneliness scale and can be considered a reliable measure of loneliness^110–112^. The respondents were asked how many days they felt lonely during the last week, with people who reportedly felt lonely one or more days being coded as lonely. All other respondents were coded as being not lonely.

### Social disengagement

We computed social disengagement based on the levels of participation in seven different social activities (Cronbach $\mathrm{\alpha }$ = 0.96). The respondents were coded as socially disengaged if they did not participate in any of the seven social activities, which included senior citizen association meetings, volunteering activities, religious service attendance, social club participation, hobby activities with others, activities in a local organization, and participation in senior job placements during the last year^9,13,113,114^.

### Group-level segregation

Group-level segregation was assessed using the complete social network of village K^115^. The KSHAP made the possible discussion network rosters include up to six people, including respondents’ spouses. The following name-generating question was used to elicit discussion network members:

“From time to time, most people discuss things that are important to them with others. For example, good or bad things that happen to you, problems you are having, or important concerns you may have. Looking back over the last 12 months, who are the people with whom you most often discussed things that were important to you?”.

The KSHAP also collected detailed information on participants’ social network members, including their names, sexes, ages, and addresses at the smallest administrative unit (the *Ri*). Based on the network roster generated, we constructed the social network map of this area. Detailed information on the method used to construct the complete social network map of village K is presented elsewhere^106^.

In social network analyses, an important component is a group of interconnected people that are disconnected from the other components. There are no social ties from one component to another. Additionally, the diameter of a component is measured as the length of the longest path (in terms of the number of ties) between two people within it. Thus, if the diameter of a component (or group) is two, everyone can reach anyone else in the group via a maximum of two paths (or ties); in other words, everyone is either a “friend” or a “friend of a friend” to one another. In our study population, the median diameter across all waves was four; thus, older adults across the entirety of this social network were coded as being group-level segregated if they belonged to a network component whose diameter was three or less. Thus, the friendships of group-level segregated people within this study were limited to up to third-degree friends: here, they would have (1) friends and (2) friends of friends (second-degree friends), and (3) friends of friends of friends (third-degree friends) arising from the group. Conversely, people who were not group-level segregated had fourth-degree or fifth-degree friends across the entirety of the village. This group-level segregation is distinct from loneliness or individual-level disengagement because it is possible for people to be group-level segregated even if they engage in numerous social activities and do not feel lonely. Those who are not group-level segregated have a friendship circle that could expand to include the whole village, while the friendship circle of those who are group-level segregated would stay within a small, distinct group. We excluded 103 isolated people with no social ties within the village from our analyses to measure the effect of group-level segregation that is independent of individual-level isolation. The correlation coefficients between these three aspects of social relationships ranged from 0.05 to 0.08.

### Control variables

Sociodemographic characteristics included sex, education, household yearly income, and living with a spouse. Living with a spouse was dichotomized as either yes or no. As the average educational level among older adults in our sample was low, their educational levels were coded as a binary: those who had graduated high school or higher were coded as 1, and all others were coded as 0. About 60% of our sample earned less than about $10,000 a year and the distribution was strongly skewed to the right. Thus, yearly income level was also coded as a binary: 1 for $10,000 or higher per year and 0 for otherwise. The measures of participants’ health statuses included comorbidity, cognitive functioning, physical health status, and depression. Following previous studies of mortality, the comorbidity measure was constructed by counting the number of diagnoses out of the following illnesses for each participant: diabetes^13,116–121^, cancer^13,119,120^, angina^116–120^, cataract^120–122^, and osteoporosis^120,121,123^. Cognitive functioning was assessed using the Korean version of the Mini Mental State Examination for Dementia Screening, with possible scores ranging from zero to thirty—higher values reflected a better cognitive health status^124^. Physical health statuses were assessed using the six-item physical component summary (PCS) from the SF-12 using standard methods^125,126^—higher scores indicated better physical health statuses. Depression was measured using the standardized CES-D items after excluding the item measuring loneliness^9,127^.

### Analytical strategy

There exist two major types of potential biases when the effects of social relationships on mortality rates are examined via a cohort (or panel) data set. First, there are potentially biased estimators due to time-varying confounders. A time-varying confounder is problematic if (1) there exists a time-varying covariate that is a risk factor for the event of interest (i.e., deaths during our study) and is also a predictor for subsequent treatment (i.e., loneliness, disengagement, or segregation) and (2) time-dependent confounders are also affected by previous treatments (i.e., loneliness, disengagement, or segregation). Subsequently, the traditional proportional hazard model for longitudinal data could produce biased estimates^128,129^. For example, deteriorating health as a time-varying factor could cause both poorer social relationships and death, in addition to the fact that health may deteriorate as a result of unsatisfactory social relationships, thus forming a circular relationship. To minimize the bias resulting from time-varying covariates, we adapted a Cox proportional marginal structural model (hereafter Cox MSM) with inverse probability weighting.

The second type of potential bias arises from the attrition of the original sample in any longitudinal data set. This attrition would produce another form of bias because the health statuses of people who leave the study could be different from those who remain in the sample—for example, respondents who leave the study tend to be sicker. To minimize biases from sample attrition, we applied censoring weights to all the study models. Inverse probability and censoring weights were truncated at the 1st and 99th percentiles to prevent influence from outlying observations^130^.

Before conducting the multivariate analyses, a series of bivariate analyses were examined; the differences in demographic and health characteristics by loneliness, social disengagement, and group-level segregation, were assessed using t-tests for continuous variables and *chi*-square tests for categorical variables. Following this, hazard ratios and 95% confidence intervals were estimated using a traditional Cox proportional hazards model (Cox PH models) to examine the associations of loneliness, social disengagement, and group-level segregation with mortality rates during the follow-up period between 2011 and 2019. Finally, a Cox MSM was utilized to minimize the biases from the reverse causations. The Cox MSM estimated both the average treatment effects (ATE) and the average treatment effects on the treated (ATT). Parameter estimates for models predicting loneliness, social disengagement, and group-level segregation are provided in supplementary material (Tables S1-S3). Additionally, the distribution of weights for the ATE and ATT models is provided in the supplementary material (Table S4). We used Stata 16.0 (StataCorp LP., College Station, TX, USA) for the statistical analyses.

## Supplementary Information

Supplementary file

## Data Availability

The data sets generated and analyzed during the current study are available in the figshare repository, 10.6084/m9.figshare.12044064.v1.
