# Supplementary material for: All-cause mortality and three aspects of social relationships: an eight-year follow-up of older adults from one entire Korean village
Source: Sci Rep. 2021 Jan 11;11:465. doi: 10.1038/s41598-020-80684-5 (PMC7801415; doi:10.1038/s41598-020-80684-5)
Supplement: Supplementary file 1 — Supplementary file [file 41598_2020_80684_MOESM1_ESM.docx]

**All-cause mortality and three aspects of social relationships: an eight-year follow-up of older adults from one entire Korean village**

Yoosik Youm^1^, Ekaterina Baldina^1^, Jiwon Baek^2^

^1^Yonsei University, Department of Sociology, Seoul, 03722, Republic of Korea

^2^Institute of Health Policy and Management, Medical Research Center, Seoul National University, Seoul, 03080, Republic of Korea

Correspondence and requests for materials should be addressed to Y.Y. (**email:** yoosik@yonsei.ac.kr.)

**Supplementary material**

Since we used an observational data set for the estimation of a causal effect of social factors on mortality, we utilized the counter-factual framework in our analysis to adjust for confounding as described by Robins et al. [1]. We estimated the marginal structural model that is designed to adjust for time-varying confounding and selection bias. Both the average treatment effect (ATE) and the average treatment on the treated (ATT) were estimated [2].

Additionally, the participants who dropped out of the study during the follow-up can cause informative censoring. Therefore, we needed to compute the weights to adjust for censoring [1] , using a logistic regression model. Weights were estimated by combining the inverse probability of treatment weights (ATEW) and censoring weights (CW). Both ATEW and CW were computed as the ratio of the estimated probabilities of treatment using the time-invariant covariates as the numerator to the estimated probabilities of treatment using the current time-variant and time-invariant (baseline) covariates as the denominator. Two logistic regressions were fitted to estimate the numerator and denominator of the ATEW. All inverse probability weights were stabilized [3]. For the case of ATT, weights were estimated by assigning the weight of 1 to the respondents who were actually experiencing loneliness (or social disengagement or group-level segregation) and assigning the weight as the ratio of probability to undergo treatment effect as the nominator to the probability not to face treatment effect as the denominator. Probabilities were estimated using the current time-variant and time-invariant (baseline) covariates. Final weights for the ATT model were estimated by combining the ATTW and CW.

The validity of the models was assessed by covariate balance checking before and after the application of weights. We computed the standardized difference in means across covariates between the treated (lonely, disengaged and segregated) group and non-treated group before and after the application of weights. Covariates are balanced if standardized mean difference in covariates between treated and non-treated group after application of weights do not exceed 0.2, threshold widely accepted by other studies [2, 4] . Detailed information on balance check for each treatment variable and each MSM model presented is in Tables S5a-S7b.

Table S1. Pooled logistic regression models predicting loneliness (N=1,483)

| Variables | Denominator | | | Nominator | | |
| --- | --- | --- | --- | --- | --- | --- |
|  | OR | p-value | 95% CI | OR | p-value | 95% CI |
| Education, ≥ high school | 0.95 | 0.807 | 0.65,1.40 | 0.84 | 0.269 | 0.62,1.14 |
| Female | 0.88 | 0.514 | 0.60,1.29 | 1.11 | 0.474 | 0.84,1.47 |
| Baseline covariates | |  |  |  |  |  |
| Loneliness | 3.56 | <0.001 | 1.99,6.36 | 1.81 | 0.005 | 1.19,2.75 |
| Social disengagement | 1.11 | 0.623 | 0.74,1.67 | 1.01 | 0.961 | 0.75,1.35 |
| Group-level segregation | 1.01 | 0.976 | 0.64,1.60 | 1.07 | 0.687 | 0.77,1.48 |
| Living with spouse | 1.67 | 0.272 | 0.67,4.16 | 0.53 | <0.001 | 0.39,0.72 |
| Yearly income | 0.84 | 0.286 | 0.62,1.15 | 0.83 | 0.131 | 0.65,1.06 |
| Smoking | 0.81 | 0.473 | 0.46,1.44 | 0.99 | 0.945 | 0.68,1.43 |
| Drinking alcohol | 1.41 | 0.126 | 0.91,2.19 | 1.35 | 0.050 | 1.00,1.84 |
| Depression | 0.37 | <0.001 | 0.23,0.60 | 1.26 | 0.069 | 0.98,1.60 |
| Physical health | 1.00 | 0.761 | 0.98,1.02 | 0.99 | 0.213 | 0.98,1.01 |
| Cognitive health | 0.99 | 0.712 | 0.95,1.03 | 0.98 | 0.106 | 0.95,1.00 |
| Comorbidity | 0.98 | 0.838 | 0.81,1.18 | 1.05 | 0.547 | 0.90,1.22 |
| Time-varying covariates (t-1) | | | |  |  |  |
| Loneliness | 3.75 | <0.001 | 2.22,6.36 | 4.66 | <0.001 | 3.17,6.86 |
| Time-varying covariates (current t) | | | |  |  |  |
| Social disengagement | 0.68 | 0.134 | 0.41,1.13 |  |  |  |
| Group-level segregation | 1.34 | 0.177 | 0.88,2.04 |  |  |  |
| Living with spouse | 0.22 | 0.001 | 0.09,0.54 |  |  |  |
| Yearly income | 0.81 | 0.240 | 0.57,1.15 |  |  |  |
| Smoking | 1.27 | 0.450 | 0.68,2.38 |  |  |  |
| Drinking alcohol | 0.94 | 0.815 | 0.58,1.54 |  |  |  |
| Depression | 22.58 | <0.001 | 13.95,36.56 |  |  |  |
| Physical health | 1.00 | 0.969 | 0.98,1.02 |  |  |  |
| Cognitive health | 1.05 | 0.015 | 1.01,1.09 |  |  |  |
| Comorbidity | 0.92 | 0.441 | 0.75,1.14 |  |  |  |

| Variables | Denominator | | | Nominator | | |
| --- | --- | --- | --- | --- | --- | --- |
|  | OR | p-value | 95% CI | OR | p-value | 95% CI |
| Education, ≥ high school | 0.50 | 0.017 | 0.28,0.88 | 0.39 | 0.001 | 0.23,0.67 |
| Female | 0.89 | 0.606 | 0.56,1.40 | 0.98 | 0.937 | 0.64,1.52 |
| Baseline covariates | |  |  |  |  |  |
| Loneliness | 0.88 | 0.583 | 0.55,1.40 | 0.83 | 0.401 | 0.54,1.28 |
| Social disengagement | 1.21 | 0.381 | 0.79,1.85 | 1.17 | 0.486 | 0.76,1.79 |
| Group-level segregation | 1.18 | 0.543 | 0.69,2.01 | 1.27 | 0.323 | 0.79,2.06 |
| Living with spouse | 0.77 | 0.455 | 0.40,1.52 | 0.63 | 0.031 | 0.41,0.96 |
| Yearly income | 1.17 | 0.403 | 0.81,1.68 | 1.09 | 0.626 | 0.77,1.54 |
| Smoking | 1.85 | 0.109 | 0.87,3.94 | 1.29 | 0.424 | 0.69,2.43 |
| Drinking alcohol | 1.51 | 0.120 | 0.90,2.53 | 1.12 | 0.649 | 0.69,1.81 |
| Depression | 0.90 | 0.668 | 0.57,1.43 | 1.08 | 0.656 | 0.76,1.55 |
| Physical health | 0.99 | 0.210 | 0.96,1.01 | 0.97 | 0.004 | 0.95,0.99 |
| Cognitive health | 1.00 | 0.931 | 0.96,1.05 | 0.95 | 0.004 | 0.91,0.98 |
| Comorbidity | 1.05 | 0.688 | 0.81,1.36 | 1.04 | 0.698 | 0.84,1.30 |
| Time-varying covariates (t-1) | | | |  |  |  |
| Social disengagement | 3.02 | <0.001 | 1.91,4.78 | 3.70 | <0.001 | 2.39,5.71 |
| Time-varying covariates (current t) | | | |  |  |  |
| Loneliness | 0.61 | 0.060 | 0.37,1.02 |  |  |  |
| Group-level segregation | 1.55 | 0.035 | 1.03,2.32 |  |  |  |
| Living with spouse | 0.84 | 0.586 | 0.45,1.57 |  |  |  |
| Yearly income | 0.76 | 0.127 | 0.54,1.08 |  |  |  |
| Smoking | 1.51 | 0.120 | 0.90,2.53 |  |  |  |
| Drinking alcohol | 0.59 | 0.082 | 0.33,1.07 |  |  |  |
| Depression | 1.54 | 0.013 | 1.10,2.17 |  |  |  |
| Physical health | 0.96 | <0.001 | 0.94,0.98 |  |  |  |
| Cognitive health | 0.93 | <0.001 | 0.90,0.96 |  |  |  |
| Comorbidity | 0.80 | 0.052 | 0.63,1.00 |  |  |  |

Table S2. Pooled logistic regression models predicting social disengagement (N=1,483)

Table S3. Pooled logistic regression models predicting group-level segregation (N=1,483)

| Variables | Denominator | | | Nominator | | |
| --- | --- | --- | --- | --- | --- | --- |
|  | OR | p-value | 95% CI | OR | p-value | 95% CI |
| Education, ≥ high school | 2.12 | <0.001 | 1.44,3.12 | 2.04 | <0.001 | 1.42,2.94 |
| Female | 1.19 | 0.347 | 0.83,1.73 | 1.27 | 0.201 | 0.88,1.83 |
| Baseline covariates | | | | | | |
| Loneliness | 0.56 | 0.004 | 0.38,0.83 | 0.67 | 0.031 | 0.46,0.96 |
| Social disengagement | 1.36 | 0.110 | 0.93,1.97 | 1.42 | 0.056 | 0.99,2.04 |
| Group-level segregation | 3.17 | <0.001 | 2.08,4.84 | 3.15 | <0.001 | 2.08,4.77 |
| Living with spouse | 0.81 | 0.677 | 0.29,2.23 | 0.94 | 0.765 | 0.61,1.43 |
| Yearly income | 1.01 | 0.941 | 0.74,1.39 | 1.06 | 0.716 | 0.78,1.44 |
| Smoking | 1.30 | 0.396 | 0.71,2.36 | 1.03 | 0.901 | 0.64,1.67 |
| Drinking alcohol | 0.96 | 0.866 | 0.58,1.59 | 0.90 | 0.638 | 0.58,1.39 |
| Depression | 1.08 | 0.666 | 0.76,1.52 | 1.14 | 0.420 | 0.83,1.58 |
| Physical health | 0.99 | 0.172 | 0.97,1.01 | 0.99 | 0.160 | 0.97,1.01 |
| Cognitive health | 0.98 | 0.230 | 0.94,1.02 | 0.97 | 0.057 | 0.93,1.00 |
| Comorbidity | 0.99 | 0.933 | 0.80,1.22 | 0.92 | 0.397 | 0.76,1.11 |
| Time-varying covariates (t-1) | | | | | | |
| Group-level segregation | 3.22 | <0.001 | 2.21,4.68 | 3.19 | <0.001 | 2.19,4.64 |
| Time-varying covariates (current t) | | | | | | |
| Loneliness | 1.59 | 0.023 | 1.07,2.38 |  |  |  |
| Social disengagement | 1.45 | 0.059 | 0.99,2.15 |  |  |  |
| Living with spouse | 1.27 | 0.620 | 0.49,3.29 |  |  |  |
| Yearly income | 1.16 | 0.371 | 0.83,1.63 |  |  |  |
| Smoking | 0.65 | 0.209 | 0.33,1.27 |  |  |  |
| Drinking alcohol | 0.71 | 0.219 | 0.41,1.22 |  |  |  |
| Depression | 1.11 | 0.497 | 0.82,1.52 |  |  |  |
| Physical health | 1.01 | 0.397 | 0.99,1.03 |  |  |  |
| Cognitive health | 0.98 | 0.441 | 0.94,1.03 |  |  |  |
| Comorbidity | 0.83 | 0.048 | 0.69,1.00 |  |  |  |

Table S4. Distribution of ATE and ATT treatment, censor, and final weights.

| Treatment variables | Minimum | 1^st^ Quartile | Median | Mean | 3^rd^ Quartile | Maximum |
| --- | --- | --- | --- | --- | --- | --- |
| *ATE weights (ATEW)* | | | | | | |
| Loneliness | 0.11 | 0.70 | 0.90 | 0.95 | 0.98 | 5.81 |
| Social disengagement | 0.38 | 0.92 | 0.98 | 0.99 | 1.03 | 2.01 |
| Group-level segregation | 0.61 | 0.95 | 0.99 | 1.00 | 1.04 | 1.59 |
| *ATT weights (ATTW)* | | | | | | |
| Loneliness | 0.00 | 0.01 | 0.11 | 0.42 | 1.00 | 3.64 |
| Social disengagement | 0.00 | 0.00 | 0.04 | 0.22 | 0.20 | 1.13 |
| Group-level segregation | 0.00 | 0.01 | 0.11 | 0.33 | 0.57 | 1.85 |
| *Censoring weights (CW)* | | | | | | |
|  | 0.81 | 0.97 | 0.99 | 1.00 | 1.02 | 1.43 |
| *Final ATE weights (ATEW x CW)* | | | | | | |
| Loneliness | 0.10 | 0.69 | 0.90 | 0.96 | 0.99 | 8.32 |
| Social disengagement | 0.32 | 0.90 | 0.97 | 0.99 | 1.04 | 2.88 |
| Group-level segregation | 0.54 | 0.93 | 0.98 | 1.00 | 1.06 | 1.88 |
| *Final ATT weights (ATTW x CW)* | | | | | | |
| Loneliness | 0.00 | 0.01 | 0.10 | 0.42 | 0.94 | 5.22 |
| Social disengagement | 0.00 | 0.00 | 0.04 | 0.23 | 0.20 | 1.62 |
| Group-level segregation | 0.00 | 0.01 | 0.11 | 0.33 | 0.60 | 2.59 |

Table S5a. Balance check for loneliness (average treatment effect weights, ATE)

| Variables | Control | | | Treatment | | | Standard. Mean Difference | | |
| --- | --- | --- | --- | --- | --- | --- | --- | --- | --- |
|  | N | Weight | Mean | N | Weight | Mean | Before | After |  |
| Time invariant variables |  |  |  |  |  |  |  |  |  |
| Education, ≥ high school | 1,087 | 1048.57 | 0.31 | 396 | 365.53 | 0.19 | 0.239 | 0.273 |  |
| Female | 1,087 | 1048.57 | 0.57 | 396 | 365.53 | 0.63 | 0.121 | 0.132 |  |
| Baseline variables |  |  |  |  |  |  |  |  |  |
| Loneliness | 1,087 | 1048.57 | 0.16 | 396 | 365.53 | 0.62 | 1.102 | 1.061 |  |
| Social disengagement | 1,087 | 1048.57 | 0.12 | 396 | 365.53 | 0.17 | 0.157 | 0.134 |  |
| Group-level segregation | 1,087 | 1048.57 | 0.18 | 396 | 365.53 | 0.20 | 0.119 | 0.034 |  |
| Living with spouse | 1,087 | 1048.57 | 0.82 | 396 | 365.53 | 0.63 | 0.444 | 0.429 |  |
| Yearly income | 1,087 | 1048.57 | 0.55 | 396 | 365.53 | 0.43 | 0.348 | 0.241 |  |
| Smoking | 1,087 | 1048.57 | 0.08 | 396 | 365.53 | 0.10 | 0.087 | 0.077 |  |
| Drinking alcohol | 1,087 | 1048.57 | 0.21 | 396 | 365.53 | 0.19 | 0.052 | 0.067 |  |
| Depression | 1,087 | 1048.57 | -0.13 | 396 | 365.53 | 0.23 | 1.352 | 0.628 |  |
| Physical health | 1,087 | 1048.57 | 46.61 | 396 | 365.53 | 42.67 | 0.394 | 0.377 |  |
| Cognitive health | 1,087 | 1048.57 | 24.73 | 396 | 365.53 | 23.22 | 0.336 | 0.313 |  |
| Comorbidity | 1,087 | 1048.57 | 2.67 | 396 | 365.53 | 2.80 | 0.166 | 0.155 |  |
| Time-variant variables |  |  |  |  |  |  |  |  |  |
| Loneliness | 1,087 | 1048.57 | 0.18 | 396 | 365.53 | 0.61 | 0.983 | 0.959 |  |
| Social disengagement | 1,087 | 1048.57 | 0.17 | 396 | 365.53 | 0.25 | 0.162 | 0.208 |  |
| Group-level segregation | 1,087 | 1048.57 | 0.13 | 396 | 365.53 | 0.17 | 0.041 | 0.100 |  |
| Living with spouse | 1,087 | 1048.57 | 0.85 | 396 | 365.53 | 0.65 | 0.361 | 0.481 |  |
| Yearly income | 1,087 | 1048.57 | 0.50 | 396 | 365.53 | 0.38 | 0.197 | 0.243 |  |
| Smoking | 1,087 | 1048.57 | 0.10 | 396 | 365.53 | 0.09 | 0.009 | 0.033 |  |
| Drinking alcohol | 1,087 | 1048.57 | 0.23 | 396 | 365.53 | 0.25 | 0.057 | 0.051 |  |
| Depression | 1,087 | 1048.57 | -0.03 | 396 | 365.53 | 0.45 | 0.677 | 0.798 |  |
| Physical health | 1,087 | 1048.57 | 47.75 | 396 | 365.53 | 43.36 | 0.420 | 0.489 |  |
| Cognitive health | 1,087 | 1048.57 | 24.67 | 396 | 365.53 | 23.14 | 0.302 | 0.340 |  |
| Comorbidity | 1,087 | 1048.57 | 2.52 | 396 | 365.53 | 2.72 | 0.238 | 0.257 |  |

Weighted means of covariates are unbalanced if standardized mean difference after weighting is higher than absolute value 0.2.

Table S5b. Balance check for loneliness (average treatment effect weights, ATT)

| Variables | Control | | | Treatment | | Standard. Mean Difference | | |  |
| --- | --- | --- | --- | --- | --- | --- | --- | --- | --- |
|  | N | Weight | Mean | N | Mean | | Before | After | |
| Time invariant variables |  |  |  |  |  | |  |  | |
| Education, ≥ high school | 1,087 | 227.85 | 0.15 | 396 | 0.22 | | 0.239 | 0.178 | |
| Female | 1,087 | 227.85 | 0.64 | 396 | 0.62 | | 0.121 | 0.053 | |
| Baseline variables |  |  |  |  |  | |  |  | |
| Loneliness | 1,087 | 227.85 | 0.58 | 396 | 0.66 | | 1.102 | 0.153 | |
| Social disengagement | 1,087 | 227.85 | 0.14 | 396 | 0.18 | | 0.157 | 0.104 | |
| Group-level segregation | 1,087 | 227.85 | 0.18 | 396 | 0.23 | | 0.119 | 0.109 | |
| Living with spouse | 1,087 | 227.85 | 0.65 | 396 | 0.65 | | 0.444 | 0.003 | |
| Yearly income | 1,087 | 227.85 | 0.43 | 396 | 0.39 | | 0.348 | 0.085 | |
| Smoking | 1,087 | 227.85 | 0.09 | 396 | 0.11 | | 0.087 | 0.094 | |
| Drinking alcohol | 1,087 | 227.85 | 0.17 | 396 | 0.20 | | 0.052 | 0.062 | |
| Depression | 1,087 | 227.85 | 0.27 | 396 | 0.51 | | 1.352 | 0.367 | |
| Physical health | 1,087 | 227.85 | 42.21 | 396 | 42.92 | | 0.394 | 0.065 | |
| Cognitive health | 1,087 | 227.85 | 23.15 | 396 | 23.19 | | 0.336 | 0.009 | |
| Comorbidity | 1,087 | 227.85 | 2.88 | 396 | 2.79 | | 0.166 | 0.106 | |
| Time-variant variables |  |  |  |  |  | |  |  | |
| Loneliness | 1,087 | 227.85 | 0.63 | 396 | 0.66 | | 0.983 | 0.053 | |
| Social disengagement | 1,087 | 227.85 | 0.25 | 396 | 0.23 | | 0.162 | 0.041 | |
| Group-level segregation | 1,087 | 227.85 | 0.13 | 396 | 0.14 | | 0.041 | 0.039 | |
| Living with spouse | 1,087 | 227.85 | 0.73 | 396 | 0.70 | | 0.361 | 0.072 | |
| Yearly income | 1,087 | 227.85 | 0.44 | 396 | 0.41 | | 0.197 | 0.059 | |
| Smoking | 1,087 | 227.85 | 0.07 | 396 | 0.12 | | 0.009 | 0.166 | |
| Drinking alcohol | 1,087 | 227.85 | 0.26 | 396 | 0.27 | | 0.057 | 0.015 | |
| Depression | 1,087 | 227.85 | 0.30 | 396 | 0.38 | | 0.677 | 0.128 | |
| Physical health | 1,087 | 227.85 | 44.25 | 396 | 43.91 | | 0.420 | 0.038 | |
| Cognitive health | 1,087 | 227.85 | 23.83 | 396 | 23.34 | | 0.302 | 0.112 | |
| Comorbidity | 1,087 | 227.85 | 2.71 | 396 | 2.74 | | 0.238 | 0.029 | |

Weighted means of covariates are unbalanced if standardized mean difference after weighting is higher than absolute value 0.2.

Table S6a. Balance check for social disengagement (average treatment effect weights, ATE)

| Variables | Control | | | Treatment | | | Standard. Mean Difference | | |
| --- | --- | --- | --- | --- | --- | --- | --- | --- | --- |
|  | N | Weight | Mean | N | Weight | Mean | Before | After |  |
| Time invariant variables |  |  |  |  |  |  |  |  |  |
| Education, ≥ high school | 1,278 | 1272.57 | 0.34 | 205 | 199.11 | 0.10 | 0.579 | 0.593 |  |
| Female | 1,278 | 1272.57 | 0.56 | 205 | 199.11 | 0.68 | 0.230 | 0.252 |  |
| Baseline variables |  |  |  |  |  |  |  |  |  |
| Loneliness | 1,278 | 1272.57 | 0.26 | 205 | 199.11 | 0.36 | 0.202 | 0.231 |  |
| Social disengagement | 1,278 | 1272.57 | 0.11 | 205 | 199.11 | 0.41 | 0.733 | 0.735 |  |
| Group-level segregation | 1,278 | 1272.57 | 0.19 | 205 | 199.11 | 0.24 | 0.213 | 0.127 |  |
| Living with spouse | 1,278 | 1272.57 | 0.82 | 205 | 199.11 | 0.60 | 0.469 | 0.483 |  |
| Yearly income | 1,278 | 1272.57 | 0.53 | 205 | 199.11 | 0.39 | 0.364 | 0.291 |  |
| Smoking | 1,278 | 1272.57 | 0.10 | 205 | 199.11 | 0.10 | 0.047 | 0.013 |  |
| Drinking alcohol | 1,278 | 1272.57 | 0.22 | 205 | 199.11 | 0.14 | 0.296 | 0.219 |  |
| Depression | 1,278 | 1272.57 | -0.07 | 205 | 199.11 | 0.20 | 0.527 | 0.424 |  |
| Physical health | 1,278 | 1272.57 | 46.54 | 205 | 199.11 | 41.62 | 0.766 | 0.465 |  |
| Cognitive health | 1,278 | 1272.57 | 24.78 | 205 | 199.11 | 21.87 | 0.808 | 0.564 |  |
| Comorbidity | 1,278 | 1272.57 | 2.68 | 205 | 199.11 | 2.71 | 0.053 | 0.038 |  |
| Time-variant variables |  |  |  |  |  |  |  |  |  |
| Loneliness | 1,278 | 1272.57 | 0.33 | 205 | 199.11 | 0.40 | 0.132 | 0.149 |  |
| Social disengagement | 1,278 | 1272.57 | 0.15 | 205 | 199.11 | 0.36 | 0.525 | 0.509 |  |
| Group-level segregation | 1,278 | 1272.57 | 0.13 | 205 | 199.11 | 0.18 | 0.075 | 0.141 |  |
| Living with spouse | 1,278 | 1272.57 | 0.84 | 205 | 199.11 | 0.65 | 0.440 | 0.452 |  |
| Yearly income | 1,278 | 1272.57 | 0.48 | 205 | 199.11 | 0.43 | 0.080 | 0.110 |  |
| Smoking | 1,278 | 1272.57 | 0.12 | 205 | 199.11 | 0.12 | 0.025 | 0.000 |  |
| Drinking alcohol | 1,278 | 1272.57 | 0.26 | 205 | 199.11 | 0.22 | 0.082 | 0.082 |  |
| Depression | 1,278 | 1272.57 | 0.06 | 205 | 199.11 | 0.32 | 0.362 | 0.395 |  |
| Physical health | 1,278 | 1272.57 | 47.46 | 205 | 199.11 | 42.12 | 0.573 | 0.567 |  |
| Cognitive health | 1,278 | 1272.57 | 24.82 | 205 | 199.11 | 21.71 | 0.636 | 0.625 |  |
| Comorbidity | 1,278 | 1272.57 | 2.58 | 205 | 199.11 | 2.74 | 0.156 | 0.187 |  |

Weighted means of covariates are unbalanced if standardized mean difference after weighting is higher than absolute value 0.2.

Table S6b. Balance check for social disengagement (average treatment effects of the treated, ATT)

| Variables | Control | | | Treatment | | Standard. Mean Difference | | | |
| --- | --- | --- | --- | --- | --- | --- | --- | --- | --- |
|  | N | Weight | Mean | N | Mean | | Before | After |  |
| Time invariant variables |  |  |  |  |  | |  |  |  |
| Education, ≥ high school | 1,278 | 124.63 | 0.10 | 205 | 0.10 | | 0.579 | 0.012 |  |
| Female | 1,278 | 124.63 | 0.66 | 205 | 0.67 | | 0.230 | 0.024 |  |
| Baseline variables |  |  |  |  |  | |  |  |  |
| Loneliness | 1,278 | 124.63 | 0.35 | 205 | 0.35 | | 0.202 | 0.002 |  |
| Social disengagement | 1,278 | 124.63 | 0.44 | 205 | 0.40 | | 0.733 | 0.067 |  |
| Group-level segregation | 1,278 | 124.63 | 0.29 | 205 | 0.27 | | 0.213 | 0.038 |  |
| Living with spouse | 1,278 | 124.63 | 0.62 | 205 | 0.61 | | 0.469 | 0.027 |  |
| Yearly income | 1,278 | 124.63 | 0.36 | 205 | 0.36 | | 0.364 | 0.002 |  |
| Smoking | 1,278 | 124.63 | 0.08 | 205 | 0.08 | | 0.047 | 0.015 |  |
| Drinking alcohol | 1,278 | 124.63 | 0.12 | 205 | 0.12 | | 0.296 | 0.018 |  |
| Depression | 1,278 | 124.63 | 0.24 | 205 | 0.25 | | 0.527 | 0.009 |  |
| Physical health | 1,278 | 124.63 | 39.85 | 205 | 38.91 | | 0.766 | 0.084 |  |
| Cognitive health | 1,278 | 124.63 | 21.11 | 205 | 20.69 | | 0.808 | 0.067 |  |
| Comorbidity | 1,278 | 124.63 | 2.64 | 205 | 2.65 | | 0.053 | 0.012 |  |
| Time-variant variables |  |  |  |  |  | |  |  |  |
| Loneliness | 1,278 | 124.63 | 0.40 | 205 | 0.39 | | 0.132 | 0.014 |  |
| Social disengagement | 1,278 | 124.63 | 0.36 | 205 | 0.38 | | 0.525 | 0.036 |  |
| Group-level segregation | 1,278 | 124.63 | 0.18 | 205 | 0.16 | | 0.075 | 0.062 |  |
| Living with spouse | 1,278 | 124.63 | 0.65 | 205 | 0.65 | | 0.440 | 0.007 |  |
| Yearly income | 1,278 | 124.63 | 0.45 | 205 | 0.44 | | 0.080 | 0.003 |  |
| Smoking | 1,278 | 124.63 | 0.10 | 205 | 0.11 | | 0.025 | 0.038 |  |
| Drinking alcohol | 1,278 | 124.63 | 0.20 | 205 | 0.22 | | 0.082 | 0.046 |  |
| Depression | 1,278 | 124.63 | 0.34 | 205 | 0.29 | | 0.362 | 0.065 |  |
| Physical health | 1,278 | 124.63 | 41.83 | 205 | 42.10 | | 0.573 | 0.028 |  |
| Cognitive health | 1,278 | 124.63 | 21.45 | 205 | 21.65 | | 0.636 | 0.035 |  |
| Comorbidity | 1,278 | 124.63 | 2.68 | 205 | 2.71 | | 0.156 | 0.033 |  |

Weighted means of covariates are unbalanced if standardized mean difference after weighting is higher than absolute value 0.2.

Table S7a. Balance check for group-level segregation (average treatment effect weights, ATE)

| Variables | Control | | | Treatment | | | Standard. Mean Difference | |  |
| --- | --- | --- | --- | --- | --- | --- | --- | --- | --- |
|  | N | Weight | Mean | N | Weight | Mean | Before | After | |
| Time invariant variables |  |  |  |  |  |  |  |  | |
| Education, ≥ high school | 1,198 | 1195.61 | 0.27 | 285 | 286.45 | 0.40 | 0.291 | 0.265 | |
| Female | 1,198 | 1195.61 | 0.57 | 285 | 286.45 | 0.61 | 0.069 | 0.080 | |
| Baseline variables |  |  |  |  |  |  |  |  | |
| Loneliness | 1,198 | 1195.61 | 0.27 | 285 | 286.45 | 0.28 | 0.134 | 0.025 | |
| Social disengagement | 1,198 | 1195.61 | 0.12 | 285 | 286.45 | 0.47 | 0.796 | 0.820 | |
| Group-level segregation | 1,198 | 1195.61 | 0.13 | 285 | 286.45 | 0.17 | 0.186 | 0.097 | |
| Living with spouse | 1,198 | 1195.61 | 0.79 | 285 | 286.45 | 0.77 | 0.005 | 0.030 | |
| Yearly income | 1,198 | 1195.61 | 0.50 | 285 | 286.45 | 0.54 | 0.134 | 0.073 | |
| Smoking | 1,198 | 1195.61 | 0.09 | 285 | 286.45 | 0.09 | 0.044 | 0.015 | |
| Drinking alcohol | 1,198 | 1195.61 | 0.21 | 285 | 286.45 | 0.20 | 0.093 | 0.026 | |
| Depression | 1,198 | 1195.61 | -0.05 | 285 | 286.45 | -0.01 | 0.144 | 0.062 | |
| Physical health | 1,198 | 1195.61 | 45.80 | 285 | 286.45 | 45.54 | 0.021 | 0.025 | |
| Cognitive health | 1,198 | 1195.61 | 24.41 | 285 | 286.45 | 24.30 | 0.077 | 0.024 | |
| Comorbidity | 1,198 | 1195.61 | 2.70 | 285 | 286.45 | 2.63 | 0.162 | 0.076 | |
| Time-variant variables |  |  |  |  |  |  |  |  | |
| Loneliness | 1,198 | 1195.61 | 0.34 | 285 | 286.45 | 0.29 | 0.071 | 0.101 | |
| Social disengagement | 1,198 | 1195.61 | 0.08 | 285 | 286.45 | 0.37 | 0.746 | 0.748 | |
| Group-level segregation | 1,198 | 1195.61 | 0.17 | 285 | 286.45 | 0.22 | 0.141 | 0.132 | |
| Living with spouse | 1,198 | 1195.61 | 0.81 | 285 | 286.45 | 0.80 | 0.022 | 0.023 | |
| Yearly income | 1,198 | 1195.61 | 0.47 | 285 | 286.45 | 0.51 | 0.102 | 0.079 | |
| Smoking | 1,198 | 1195.61 | 0.11 | 285 | 286.45 | 0.11 | 0.021 | 0.011 | |
| Drinking alcohol | 1,198 | 1195.61 | 0.25 | 285 | 286.45 | 0.22 | 0.062 | 0.064 | |
| Depression | 1,198 | 1195.61 | 0.09 | 285 | 286.45 | 0.10 | 0.030 | 0.019 | |
| Physical health | 1,198 | 1195.61 | 46.74 | 285 | 286.45 | 46.13 | 0.053 | 0.069 | |
| Cognitive health | 1,198 | 1195.61 | 24.50 | 285 | 286.45 | 23.61 | 0.162 | 0.176 | |
| Comorbidity | 1,198 | 1195.61 | 2.61 | 285 | 286.45 | 2.51 | 0.120 | 0.130 | |

Weighted means of covariates are unbalanced if standardized mean difference after weighting is higher than absolute value 0.2.

Table S7b. Balance check for group-level segregation (average treatment effect of the treated weights, ATT)

| Variables | Control | | | Treatment | | Standard. Mean Difference | |
| --- | --- | --- | --- | --- | --- | --- | --- |
|  | N | Weight | Mean | N | Mean | Before | After |
| Time invariant variables |  |  |  |  |  |  |  |
| Education, ≥ high school | 1,198 | 204.54 | 0.43 | 285 | 0.41 | 0.291 | 0.048 |
| Female | 1,198 | 204.54 | 0.61 | 285 | 0.60 | 0.069 | 0.013 |
| Baseline variables |  |  |  |  |  |  |  |
| Loneliness | 1,198 | 204.54 | 0.35 | 285 | 0.32 | 0.134 | 0.063 |
| Social disengagement | 1,198 | 204.54 | 0.54 | 285 | 0.46 | 0.796 | 0.173 |
| Group-level segregation | 1,198 | 204.54 | 0.20 | 285 | 0.19 | 0.186 | 0.013 |
| Living with spouse | 1,198 | 204.54 | 0.79 | 285 | 0.79 | 0.005 | 0.017 |
| Yearly income | 1,198 | 204.54 | 0.55 | 285 | 0.57 | 0.134 | 0.035 |
| Smoking | 1,198 | 204.54 | 0.08 | 285 | 0.08 | 0.044 | 0.004 |
| Drinking alcohol | 1,198 | 204.54 | 0.17 | 285 | 0.18 | 0.093 | 0.022 |
| Depression | 1,198 | 204.54 | 0.06 | 285 | 0.02 | 0.144 | 0.053 |
| Physical health | 1,198 | 204.54 | 45.56 | 285 | 46.07 | 0.021 | 0.048 |
| Cognitive health | 1,198 | 204.54 | 23.56 | 285 | 24.09 | 0.077 | 0.089 |
| Comorbidity | 1,198 | 204.54 | 2.61 | 285 | 2.58 | 0.162 | 0.044 |
| Time-variant variables |  |  |  |  |  |  |  |
| Loneliness | 1,198 | 204.54 | 0.39 | 285 | 0.31 | 0.071 | 0.167 |
| Social disengagement | 1,198 | 204.54 | 0.44 | 285 | 0.37 | 0.746 | 0.147 |
| Group-level segregation | 1,198 | 204.54 | 0.25 | 285 | 0.23 | 0.141 | 0.043 |
| Living with spouse | 1,198 | 204.54 | 0.80 | 285 | 0.80 | 0.022 | 0.006 |
| Yearly income | 1,198 | 204.54 | 0.55 | 285 | 0.52 | 0.102 | 0.067 |
| Smoking | 1,198 | 204.54 | 0.10 | 285 | 0.12 | 0.021 | 0.057 |
| Drinking alcohol | 1,198 | 204.54 | 0.22 | 285 | 0.23 | 0.062 | 0.025 |
| Depression | 1,198 | 204.54 | 0.19 | 285 | 0.11 | 0.030 | 0.137 |
| Physical health | 1,198 | 204.54 | 45.83 | 285 | 46.29 | 0.053 | 0.052 |
| Cognitive health | 1,198 | 204.54 | 23.40 | 285 | 23.69 | 0.162 | 0.051 |
| Comorbidity | 1,198 | 204.54 | 2.49 | 285 | 2.52 | 0.120 | 0.029 |

Weighted means of covariates are unbalanced if standardized mean difference after weighting is higher than absolute value 0.2.

Table S8. Cox PH model with loneliness as the main predictor with different health time-varying mediators (n=679)

| Variables | Baseline | | | w/o TV | | | | Depression (TV) | | | | Physical health (TV) | | | | Cognitive health (TV) | | | |
| --- | --- | --- | --- | --- | --- | --- | --- | --- | --- | --- | --- | --- | --- | --- | --- | --- | --- | --- | --- |
|  | HR | 95%CI | p-value | HR | 95%CI | p-value | HR | | 95%CI | p-value | HR | | 95%CI | p-value | HR | | 95%CI | p-value |  |
| Loneliness | 1.88 | 1.11,3.17 | 0.019 | 1.80 | 1.08,3.03 | 0.025 | 1.31 | | 0.68,2.51 | 0.417 | 1.76 | | 1.04,2.99 | 0.036 | 1.78 | | 1.06,2.98 | 0.029 |  |
| Education  (high school or higher) | 1.02 | 0.46,2.29 | 0.953 | 0.93 | 0.42,2.07 | 0.859 | 0.89 | | 0.41,1.98 | 0.783 | 0.94 | | 0.43,2.07 | 0.878 | 0.96 | | 0.43,2.15 | 0.915 |  |
| Female | 0.46 | 0.25,0.86 | 0.015 | 0.39 | 0.21,0.74 | 0.004 | 0.36 | | 0.19,0.68 | 0.002 | 0.39 | | 0.21,0.74 | 0.004 | 0.39 | | 0.21,0.74 | 0.004 |  |
| **Baseline covariates** |  |  |  |  |  |  |  | |  |  |  | |  |  |  | |  |  |  |
| Loneliness | 0.87 | 0.49,1.52 | 0.618 | 0.78 | 0.44,1.39 | 0.400 | 0.87 | | 0.48,1.57 | 0.643 | 0.75 | | 0.42,1.34 | 0.334 | 0.78 | | 0.44,1.38 | 0.388 |  |
| Social disengagement | 0.89 | 0.46,1.72 | 0.735 | 0.68 | 0.35,1.31 | 0.246 | 0.67 | | 0.34,1.31 | 0.239 | 0.67 | | 0.35,1.29 | 0.236 | 0.65 | | 0.33,1.27 | 0.207 |  |
| Group-level segregation | 1.16 | 0.54,2.50 | 0.702 | 0.96 | 0.44,2.12 | 0.920 | 0.97 | | 0.44,2.12 | 0.933 | 0.98 | | 0.45,2.14 | 0.957 | 1.01 | | 0.45,2.23 | 0.986 |  |
| Living with spouse | 1.05 | 0.53,2.06 | 0.898 | 1.13 | 0.37,3.39 | 0.831 | 0.97 | | 0.30,3.08 | 0.953 | 1.11 | | 0.36,3.45 | 0.852 | 1.20 | | 0.39,3.70 | 0.754 |  |
| Yearly income | 0.93 | 0.53,1.61 | 0.785 | 0.79 | 0.45,1.41 | 0.430 | 0.78 | | 0.43,1.42 | 0.421 | 0.82 | | 0.47,1.46 | 0.507 | 0.82 | | 0.46,1.47 | 0.512 |  |
| Smoking | 1.51 | 0.76,3.00 | 0.236 | 1.64 | 0.84,3.19 | 0.145 | 1.70 | | 0.86,3.33 | 0.125 | 1.68 | | 0.87,3.27 | 0.125 | 1.82 | | 0.94,3.52 | 0.076 |  |
| Drinking alcohol | 0.79 | 0.42,1.49 | 0.464 | 0.79 | 0.39,1.63 | 0.528 | 0.76 | | 0.38,1.55 | 0.455 | 0.80 | | 0.40,1.62 | 0.537 | 0.82 | | 0.40,1.69 | 0.592 |  |
| Depression | 1.09 | 0.67,1.79 | 0.730 | 1.14 | 0.70,1.86 | 0.606 | 0.88 | | 0.53,1.46 | 0.621 | 1.13 | | 0.69,1.85 | 0.617 | 1.13 | | 0.67,1.89 | 0.643 |  |
| Physical health | 0.97 | 0.94,1.00 | 0.083 | 0.98 | 0.95,1.01 | 0.191 | 0.98 | | 0.95,1.01 | 0.156 | 0.98 | | 0.95,1.02 | 0.329 | 0.98 | | 0.95,1.01 | 0.226 |  |
| Cognitive health | 0.98 | 0.91,1.06 | 0.625 | 1.00 | 0.92,1.08 | 0.943 | 1.01 | | 0.92,1.10 | 0.910 | 1.00 | | 0.92,1.08 | 0.974 | 1.01 | | 0.93,1.09 | 0.892 |  |
| Comorbidity | 0.90 | 0.63,1.29 | 0.571 | 0.79 | 0.55,1.12 | 0.187 | 0.77 | | 0.54,1.11 | 0.162 | 0.79 | | 0.55,1.13 | 0.192 | 0.78 | | 0.55,1.11 | 0.169 |  |
| **Time-varying covariates** | |  |  |  |  |  |  | |  |  |  | |  |  |  | |  |  |  |
| Social disengagement |  |  |  | 2.14 | 1.21,3.79 | 0.009 | 1.98 | | 1.12,3.51 | 0.019 | 1.93 | | 1.08,3.47 | 0.027 | 2.00 | | 1.14,3.50 | 0.016 |  |
| Group-level segregation |  |  |  | 1.89 | 1.07,3.34 | 0.029 | 1.91 | | 1.07,3.40 | 0.029 | 1.83 | | 1.03,3.26 | 0.040 | 1.79 | | 1.00,3.19 | 0.048 |  |
| Living with a spouse |  |  |  | 0.95 | 0.33,2.73 | 0.928 | 1.05 | | 0.35,3.09 | 0.933 | 0.97 | | 0.33,2.87 | 0.958 | 0.96 | | 0.33,2.78 | 0.933 |  |
| Yearly income |  |  |  | 1.19 | 0.69,2.05 | 0.541 | 1.21 | | 0.70,2.10 | 0.498 | 1.20 | | 0.69,2.07 | 0.520 | 1.10 | | 0.61,1.98 | 0.759 |  |
| Smoking |  |  |  | 0.79 | 0.37,1.66 | 0.528 | 0.75 | | 0.35,1.58 | 0.442 | 0.86 | | 0.40,1.84 | 0.696 | 0.70 | | 0.32,1.52 | 0.370 |  |
| Drinking alcohol |  |  |  | 0.91 | 0.38,2.19 | 0.835 | 0.93 | | 0.39,2.23 | 0.869 | 0.95 | | 0.40,2.26 | 0.907 | 0.91 | | 0.38,2.18 | 0.831 |  |
| Comorbidity |  |  |  | 1.47 | 1.08,2.01 | 0.015 | 1.46 | | 1.08,1.98 | 0.015 | 1.43 | | 1.05,1.94 | 0.022 | 1.48 | | 1.09,2.02 | 0.012 |  |
| Depression |  |  |  |  |  |  | 1.59 | | 1.00,2.53 | 0.049 |  | |  |  |  | |  |  |  |
| Physical health |  |  |  |  |  |  |  | |  |  | 0.98 | | 0.95,1.01 | 0.237 |  | |  |  |  |
| Cognitive health |  |  |  |  |  |  |  | |  |  |  | |  |  | 0.97 | | 0.93,1.02 | 0.233 |  |

Exponentiated coefficients. TV – time-varying covariate; HR – Hazards Ratios; 95%CI - 95% Confidence Interval

Table S9. Cox PH model with social disengagement as the main predictor with different health time-varying mediators (N=679)

| **Variables** | Baseline | | | w/o TV | | | Depression (TV) | | | Physical health (TV) | | | Cognitive health (TV) | | |
| --- | --- | --- | --- | --- | --- | --- | --- | --- | --- | --- | --- | --- | --- | --- | --- |
|  | HR | 95%CI | p-value | HR | 95%CI | p-value | HR | 95%CI | p-value | HR | 95%CI | p-value | HR | 95%CI | p-value |
| Social disengagement | 2.09 | 1.21,3.62 | 0.008 | 2.14 | 1.21,3.79 | 0.009 | 1.98 | 1.12,3.51 | 0.019 | 1.93 | 1.08,3.47 | 0.027 | 2.00 | 1.14,3.50 | 0.016 |
| Education, ≥ high school | 1.07 | 0.48,2.38 | 0.871 | 0.93 | 0.42,2.07 | 0.859 | 0.89 | 0.41,1.98 | 0.783 | 0.94 | 0.43,2.07 | 0.878 | 0.96 | 0.43,2.15 | 0.915 |
| Female | 0.48 | 0.26,0.89 | 0.020 | 0.39 | 0.21,0.74 | 0.004 | 0.36 | 0.19,0.68 | 0.002 | 0.39 | 0.21,0.74 | 0.004 | 0.39 | 0.21,0.74 | 0.004 |
| **Baseline covariates** |  |  |  |  |  |  |  |  |  |  |  |  |  |  |  |
| Loneliness | 0.92 | 0.50,1.68 | 0.781 | 0.78 | 0.44,1.39 | 0.400 | 0.87 | 0.48,1.57 | 0.643 | 0.75 | 0.42,1.34 | 0.334 | 0.78 | 0.44,1.38 | 0.388 |
| Social disengagement | 0.74 | 0.40,1.40 | 0.357 | 0.68 | 0.35,1.31 | 0.246 | 0.67 | 0.34,1.31 | 0.239 | 0.67 | 0.35,1.29 | 0.236 | 0.65 | 0.33,1.27 | 0.207 |
| Group-level segregation | 1.15 | 0.54,2.43 | 0.716 | 0.96 | 0.44,2.12 | 0.920 | 0.97 | 0.44,2.12 | 0.933 | 0.98 | 0.45,2.14 | 0.957 | 1.01 | 0.45,2.23 | 0.986 |
| Living with spouse | 1.03 | 0.53,2.00 | 0.922 | 1.13 | 0.37,3.39 | 0.831 | 0.97 | 0.30,3.08 | 0.953 | 1.11 | 0.36,3.45 | 0.852 | 1.20 | 0.39,3.70 | 0.754 |
| Yearly income | 0.83 | 0.47,1.48 | 0.532 | 0.79 | 0.45,1.41 | 0.430 | 0.78 | 0.43,1.42 | 0.421 | 0.82 | 0.47,1.46 | 0.507 | 0.82 | 0.46,1.47 | 0.512 |
| Smoking | 1.48 | 0.74,2.94 | 0.268 | 1.64 | 0.84,3.19 | 0.145 | 1.70 | 0.86,3.33 | 0.125 | 1.68 | 0.87,3.27 | 0.125 | 1.82 | 0.94,3.52 | 0.076 |
| Drinking alcohol | 0.77 | 0.41,1.44 | 0.412 | 0.79 | 0.39,1.63 | 0.528 | 0.76 | 0.38,1.55 | 0.455 | 0.80 | 0.40,1.62 | 0.537 | 0.82 | 0.40,1.69 | 0.592 |
| Depression | 1.20 | 0.72,2.00 | 0.494 | 1.14 | 0.70,1.86 | 0.606 | 0.88 | 0.53,1.46 | 0.621 | 1.13 | 0.69,1.85 | 0.617 | 1.13 | 0.67,1.89 | 0.643 |
| Physical health | 0.97 | 0.94,1.01 | 0.135 | 0.98 | 0.95,1.01 | 0.191 | 0.98 | 0.95,1.01 | 0.156 | 0.98 | 0.95,1.02 | 0.329 | 0.98 | 0.95,1.01 | 0.226 |
| Cognitive health | 0.99 | 0.91,1.07 | 0.788 | 1.00 | 0.92,1.08 | 0.943 | 1.01 | 0.92,1.10 | 0.910 | 1.00 | 0.92,1.08 | 0.974 | 1.01 | 0.93,1.09 | 0.892 |
| Comorbidity | 0.91 | 0.64,1.31 | 0.625 | 0.79 | 0.55,1.12 | 0.187 | 0.77 | 0.54,1.11 | 0.162 | 0.79 | 0.55,1.13 | 0.192 | 0.78 | 0.55,1.11 | 0.169 |
| **Time-varying covariates** | |  |  |  |  |  |  |  |  |  |  |  |  |  |  |
| Loneliness |  |  |  | 1.80 | 1.08,3.03 | 0.025 | 1.31 | 0.68,2.51 | 0.417 | 1.76 | 1.04,2.99 | 0.036 | 1.78 | 1.06,2.98 | 0.029 |
| Group-level segregation |  |  |  | 1.89 | 1.07,3.34 | 0.029 | 1.91 | 1.07,3.40 | 0.029 | 1.83 | 1.03,3.26 | 0.040 | 1.79 | 1.00,3.19 | 0.048 |
| Living with a spouse |  |  |  | 0.95 | 0.33,2.73 | 0.928 | 1.05 | 0.35,3.09 | 0.933 | 0.97 | 0.33,2.87 | 0.958 | 0.96 | 0.33,2.78 | 0.933 |
| Yearly income |  |  |  | 1.19 | 0.69,2.05 | 0.541 | 1.21 | 0.70,2.10 | 0.498 | 1.20 | 0.69,2.07 | 0.520 | 1.10 | 0.61,1.98 | 0.759 |
| Smoking |  |  |  | 0.79 | 0.37,1.66 | 0.528 | 0.75 | 0.35,1.58 | 0.442 | 0.86 | 0.40,1.84 | 0.696 | 0.70 | 0.32,1.52 | 0.370 |
| Drinking alcohol |  |  |  | 0.91 | 0.38,2.19 | 0.835 | 0.93 | 0.39,2.23 | 0.869 | 0.95 | 0.40,2.26 | 0.907 | 0.91 | 0.38,2.18 | 0.831 |
| Comorbidity |  |  |  | 1.47 | 1.08,2.01 | 0.015 | 1.46 | 1.08,1.98 | 0.015 | 1.43 | 1.05,1.94 | 0.022 | 1.48 | 1.09,2.02 | 0.012 |
| Depression |  |  |  |  |  |  | 1.59 | 1.00,2.53 | 0.049 |  |  |  |  |  |  |
| Physical health |  |  |  |  |  |  |  |  |  | 0.98 | 0.95,1.01 | 0.237 |  |  |  |
| Cognitive health |  |  |  |  |  |  |  |  |  |  |  |  | 0.97 | 0.93,1.02 | 0.233 |

Exponentiated coefficients. TV – time-varying covariate; HR – Hazards Ratios; 95%CI - 95% Confidence Intervals.

Table S10. Cox PH model with group-level segregation as the main predictor with different health time-varying mediators (N=679)

| **Variables** | Baseline | | | w/o TV | | | Depression (TV) | | | Physical health (TV) | | | Cognitive health (TV) | | |
| --- | --- | --- | --- | --- | --- | --- | --- | --- | --- | --- | --- | --- | --- | --- | --- |
|  | HR | 95%CI | p-value | HR | 95%CI | p-value | HR | 95%CI | p-value | HR | 95%CI | p-value | HR | 95%CI | p-value |
| Group-level segregation | 2.21 | 1.27,3.85 | 0.005 | 1.89 | 1.07,3.34 | 0.029 | 1.91 | 1.07,3.40 | 0.029 | 1.83 | 1.03,3.26 | 0.040 | 1.79 | 1.00,3.19 | 0.048 |
| Education, ≥ high school | 0.92 | 0.42,2.00 | 0.828 | 0.93 | 0.42,2.07 | 0.859 | 0.89 | 0.41,1.98 | 0.783 | 0.94 | 0.43,2.07 | 0.878 | 0.96 | 0.43,2.15 | 0.915 |
| Female | 0.46 | 0.25,0.86 | 0.014 | 0.39 | 0.21,0.74 | 0.004 | 0.36 | 0.19,0.68 | 0.002 | 0.39 | 0.21,0.74 | 0.004 | 0.39 | 0.21,0.74 | 0.004 |
| **Baseline covariates** |  |  |  |  |  |  |  |  |  |  |  |  |  |  |  |
| Loneliness | 1.03 | 0.58,1.81 | 0.926 | 0.78 | 0.44,1.39 | 0.400 | 0.87 | 0.48,1.57 | 0.643 | 0.75 | 0.42,1.34 | 0.334 | 0.78 | 0.44,1.38 | 0.388 |
| Social disengagement | 0.83 | 0.44,1.54 | 0.548 | 0.68 | 0.35,1.31 | 0.246 | 0.67 | 0.34,1.31 | 0.239 | 0.67 | 0.35,1.29 | 0.236 | 0.65 | 0.33,1.27 | 0.207 |
| Group-level segregation | 0.94 | 0.42,2.10 | 0.875 | 0.96 | 0.44,2.12 | 0.920 | 0.97 | 0.44,2.12 | 0.933 | 0.98 | 0.45,2.14 | 0.957 | 1.01 | 0.45,2.23 | 0.986 |
| Living with spouse | 1.01 | 0.51,1.97 | 0.986 | 1.13 | 0.37,3.39 | 0.831 | 0.97 | 0.30,3.08 | 0.953 | 1.11 | 0.36,3.45 | 0.852 | 1.20 | 0.39,3.70 | 0.754 |
| Yearly income | 0.90 | 0.52,1.58 | 0.725 | 0.79 | 0.45,1.41 | 0.430 | 0.78 | 0.43,1.42 | 0.421 | 0.82 | 0.47,1.46 | 0.507 | 0.82 | 0.46,1.47 | 0.512 |
| Smoking | 1.65 | 0.86,3.14 | 0.129 | 1.64 | 0.84,3.19 | 0.145 | 1.70 | 0.86,3.33 | 0.125 | 1.68 | 0.87,3.27 | 0.125 | 1.82 | 0.94,3.52 | 0.076 |
| Drinking alcohol | 0.77 | 0.42,1.43 | 0.411 | 0.79 | 0.39,1.63 | 0.528 | 0.76 | 0.38,1.55 | 0.455 | 0.80 | 0.40,1.62 | 0.537 | 0.82 | 0.40,1.69 | 0.592 |
| Depression | 1.13 | 0.71,1.80 | 0.611 | 1.14 | 0.70,1.86 | 0.606 | 0.88 | 0.53,1.46 | 0.621 | 1.13 | 0.69,1.85 | 0.617 | 1.13 | 0.67,1.89 | 0.643 |
| Physical health | 0.97 | 0.94,1.00 | 0.051 | 0.98 | 0.95,1.01 | 0.191 | 0.98 | 0.95,1.01 | 0.156 | 0.98 | 0.95,1.02 | 0.329 | 0.98 | 0.95,1.01 | 0.226 |
| Cognitive health | 0.99 | 0.93,1.07 | 0.857 | 1.00 | 0.92,1.08 | 0.943 | 1.01 | 0.92,1.10 | 0.910 | 1.00 | 0.92,1.08 | 0.974 | 1.01 | 0.93,1.09 | 0.892 |
| Comorbidity | 0.92 | 0.65,1.30 | 0.633 | 0.79 | 0.55,1.12 | 0.187 | 0.77 | 0.54,1.11 | 0.162 | 0.79 | 0.55,1.13 | 0.192 | 0.78 | 0.55,1.11 | 0.169 |
| **Time-varying covariates** | |  |  |  |  |  |  |  |  |  |  |  |  |  |  |
| Loneliness |  |  |  | 1.80 | 1.08,3.03 | 0.025 | 1.31 | 0.68,2.51 | 0.417 | 1.76 | 1.04,2.99 | 0.036 | 1.78 | 1.06,2.98 | 0.029 |
| Social disengagement |  |  |  | 2.14 | 1.21,3.79 | 0.009 | 1.98 | 1.12,3.51 | 0.019 | 1.93 | 1.08,3.47 | 0.027 | 2.00 | 1.14,3.50 | 0.016 |
| Living with a spouse |  |  |  | 0.95 | 0.33,2.73 | 0.928 | 1.05 | 0.35,3.09 | 0.933 | 0.97 | 0.33,2.87 | 0.958 | 0.96 | 0.33,2.78 | 0.933 |
| Yearly income |  |  |  | 1.19 | 0.69,2.05 | 0.541 | 1.21 | 0.70,2.10 | 0.498 | 1.20 | 0.69,2.07 | 0.520 | 1.10 | 0.61,1.98 | 0.759 |
| Smoking |  |  |  | 0.79 | 0.37,1.66 | 0.528 | 0.75 | 0.35,1.58 | 0.442 | 0.86 | 0.40,1.84 | 0.696 | 0.70 | 0.32,1.52 | 0.370 |
| Drinking alcohol |  |  |  | 0.91 | 0.38,2.19 | 0.835 | 0.93 | 0.39,2.23 | 0.869 | 0.95 | 0.40,2.26 | 0.907 | 0.91 | 0.38,2.18 | 0.831 |
| Comorbidity |  |  |  | 1.47 | 1.08,2.01 | 0.015 | 1.46 | 1.08,1.98 | 0.015 | 1.43 | 1.05,1.94 | 0.022 | 1.48 | 1.09,2.02 | 0.012 |
| Depression |  |  |  |  |  |  | 1.59 | 1.00,2.53 | 0.049 |  |  |  |  |  |  |
| Physical health |  |  |  |  |  |  |  |  |  | 0.98 | 0.95,1.01 | 0.237 |  |  |  |
| Cognitive health |  |  |  |  |  |  |  |  |  |  |  |  | 0.97 | 0.93,1.02 | 0.233 |

Exponentiated coefficients. TV – time-varying covariate; HR – Hazards Ratios; 95%CI - 95% Confidence Intervals.

**References**

1. Robins J.M., Hernan M.A., & Brumback B., Marginal structural models and causal inference in epidemiology. (LWW, 2000).

2. Cole S.R. & Hernán M.A., Constructing inverse probability weights for marginal structural models. *Am J Epidemiol* **168**, 656-664 (2008).

3. Cohen J., *Statistical power analysis for the behavioral sciences*. (Routledge, 2013).

4. Austin P.C., Balance diagnostics for comparing the distribution of baseline covariates between treatment groups in propensity-score matched samples. *Statistics in medicine* **28**, 3083-3107 (2009).
